# The application of mixed reality navigation system in laparoscopic partial nephrectomy for highly complex renal tumors (RENAL score ≥10): a retrospective cohort study

**DOI:** 10.1097/JS9.0000000000001983

**Published:** 2024-08-02

**Authors:** Wangmin Liu, Yanlong Wang, Zhitong Wang, Zhiqiang Cao, Yufei Yu, Jianfeng Wang, Jianbin Bi, Lina Wu, Mo Zhang

**Affiliations:** a Department of Urology, The First Hospital of China Medical University; b Institute of Urology, China Medical University; c Department of Urology, Shengjing Hospital of China Medical University, Shenyang; d Department of Urology, Second Affiliated Hospital of Dalian Medical University, Dalian; e Department of Laboratory Medicine, Shengjing Hospital of China Medical University, Shenyang, People’s Republic of China

Highlights

- Mixed reality (MR) technology shows great value in laparoscopic partial nephrectomy for highly complex renal tumors.
- MR technology improves surgical precision in tumor resection and renal reconstruction.
- MR navigation enhances perioperative outcomes and optimizes functional recovery.

Renal cell carcinoma (RCC) is the third most prevalent genitourinary cancer, accounting for 5 and 3% of all cancer cases in males and females, respectively^1^. With the advancements in surgical technology and laparoscopic equipment, partial nephrectomy (PN) has been increasingly used to treat even complex renal tumors^2^. Ensuring the safe and effective management of complex renal tumors during PN procedures has emerged as an urgent critical challenge to be addressed^3^. Mixed reality (MR) represents a novel holographic visualization technology, which has been extensively applied in various surgical procedures^4^. In this study, we aimed to evaluate the efficacy and advantages of MR navigation system in laparoscopic partial nephrectomy (LPN) for highly complex renal tumors (RENAL score ≥10) and compare the safety, functional outcomes, and oncological outcomes between MR-assisted LPN (MRLPN) and standard LPN (SLPN).

Patients diagnosed with highly complex renal tumors at our institution were consecutively enrolled in the study from October 2020 to March 2024. A retrospective analysis of the patients was performed, as shown in Supplementary Figure 1 (Supplemental Digital Content 1, http://links.lww.com/JS9/D232). For all cases, based on surgeon’s expertise and preference, either MRLPN or SLPN was performed. All LPN procedures were performed by the same experienced surgeon. The study was approved by the Institutional Ethics Committee of our hospital and conducted in compliance with the Declaration of Helsinki. We obtained informed consent from each patient. This study has been reported in accordance with strengthening the reporting of cohort, cross-sectional, and case–control studies in surgery (STROCSS) standards^5^ (Supplemental Digital Content 2, http://links.lww.com/JS9/D233). Patients in the MRLPN group underwent three-dimensional (3D) MR model rendering (Supplementary Figure 2, Supplemental Digital Content 1, http://links.lww.com/JS9/D232). Intraoperatively, the MR model was overlaid with real-time laparoscopic imaging to guide the operation (Supplementary Figure 3, Supplemental Digital Content 1, http://links.lww.com/JS9/D232). More details regarding methodology were shown in Supplementary Methods (Supplemental Digital Content 1, http://links.lww.com/JS9/D232).

A total of 108 patients were enrolled in this study, with 48 patients in the MRLPN group and 60 patients in the SLPN group. Demographic and clinical characteristics were comparable between the two groups (all *P*>0.05) (Supplementary Table 1, Supplemental Digital Content 1, http://links.lww.com/JS9/D232). The MRLPN group exhibited a significantly shorter warm ischemia time (WIT), reduced estimated blood loss (EBL), and a shorter operation time (OT) than the SLPN group (all *P*<0.05). The proportion of selective ischemia in the MRLPN group was higher than in the SLPN group (*P*=0.006). There were no significant differences between the two groups in terms of transfusion rate (*P*=0.293) and positive surgical margin rate (*P*=0.631). The MRLPN group had a lower incidence of postoperative complications (*P*=0.046) and a higher trifecta achievement rate (*P*=0.025) compared to the SLPN group. Regarding functional outcomes, the MRLPN group had a significantly lower reduction in estimated glomerular filtration rate (*P*=0.002). Additionally, the MRLPN group had a shorter length of postoperative hospital stay compared to the SLPN group (*P*<0.001) (Table 1). At last, univariate and multivariate logistic regression models (LRMs) identified MR navigation and tumor size as significant predictors of trifecta achievement (all *P*<0.05) (Table 2). No cancer-specific deaths were observed during the median follow-up of 28 months, with comparable recurrence-free survival rates between the two groups (*P*=0.307) (Supplementary Figure 4, Supplemental Digital Content 1, http://links.lww.com/JS9/D232).

**Table 1:** Comparison of patients’ perioperative outcomes between the MRLPN and SLPN groups.

Variables	MRLPN group	SLPN group	*P*
Surgical approach, *n* (%)			0.694
Transperitoneal	27 (56.3)	36 (60.0)	
Retroperitoneal	21 (43.8)	24 (40.0)	
OT (min), mean (SD)	146.6 (18.4)	164.7 (21.7)	<0.001
WIT (min), median (IQR)	25.5 (23.0–28.0)	31.0 (27.3–33.8)	<0.001^a^
Hilar clamping strategy, *n* (%)			0.006
Global ischemia	33 (68.8)	54 (90.0)	
Selective ischemia	15 (31.3)	6 (10.0)	
EBL (ml), median (IQR)	151.0 (122.0–189.5)	183.0 (149.5–227.5)	0.004^a^
Conversion to open surgery, *n* (%)	0 (0.0)	0 (0.0)	1.000^b^
Conversion to RN, *n* (%)	0 (0.0)	0 (0.0)	1.000^b^
Transfusion rate, *n* (%)	2 (4.2)	7 (11.7)	0.293^b^
PSM, *n* (%)	2 (4.2)	5 (8.3)	0.631^b^
Postoperative complications, *n* (%)	6 (12.5)	17 (28.3)	0.046
Urinary fistula	2 (4.2)	5 (8.3)	0.631^b^
Hemorrhage need SAE	1 (2.1)	4 (6.7)	0.506^b^
Postoperative hematoma	3 (6.3)	8 (13.3)	0.374^b^
Trifecta achievement, *n* (%)	20 (41.7)	13 (21.7)	0.025
LOS (d), median (IQR)	5 (4–6)	6 (5–7)	<0.001^a^
eGFR decrease (ml/min/1.73 m^2^), median (IQR)	11.4 (7.8–15.6)	17.2 (9.6–25.8)	0.002^a^

EBL, estimated blood loss; eGFR, estimated glomerular filtration rate; IQR, interquartile range; LOS, length of postoperative hospital stay; LPN, laparoscopic partial nephrectomy; MRLPN, mixed reality–assisted LPN; OT, operation time; PSM, positive surgical margin; RN, radical nephrectomy; SAE, selective renal-artery embolization; SLPN, standard LPN; WIT, warm ischemia time.

a Mann–Whitney *U*-test.

b Fisher’s exact test.

**Table 2:** Univariate and multivariate logistic regression models for predicting trifecta achievement.

	Univariate analysis		Multivariate analysis	
Variables	OR (95% CI)	*P*	OR (95% CI)	*P*
Age	0.982 (0.941–1.025)	0.406	1.068 (0.947–1.205)	0.283
BMI	1.015 (0.786–1.310)	0.911	1.123 (0.744–1.694)	0.581
Sex				
Male vs female	0.708 (0.311–1.615)	0.412	0.729 (0.197–2.691)	0.635
CCI age-adjusted	0.713 (0.461–1.105)	0.130	0.367 (0.112–1.201)	0.098
Tumor size	0.470 (0.332–0.665)	<0.001	0.387 (0.246–0.610)	<0.001
RENAL score	0.401 (0.168–0.958)	0.040	1.516 (0.444–5.177)	0.507
Tumor side				
Left vs right	1.035 (0.456–2.347)	0.935	0.676 (0.234–1.950)	0.468
Surgical approach				
Retro vs transperitoneal	0.873 (0.379–2.013)	0.751	0.521 (0.171–1.588)	0.252
MR navigation				
With vs without	2.582 (1.114–5.985)	0.027	4.248 (1.427–12.646)	0.009

CCI, Charlson Comorbidity Index; MR, mixed reality; OR, odds ratio.

Our study found that the high-fidelity 3D reconstruction for renal vasculature enabled the surgeon to accurately identify renal arteries and branch vessels supplying the tumor, resulting in a remarkably higher rate of successful selective clamping. By adjusting the transparency of renal parenchyma in the MR model intraoperatively, it permitted the surgeon to visualize the tumor hidden in the parenchyma, therefore facilitating rapid and accurate localization of renal tumors, particularly in endophytic lesions. Meanwhile, it facilitated the identification of blood vessels and collecting system surrounding the tumor, which turned to reduce the risk of injury.

During the resection phase, the step-by-step guidance of MR images facilitated an excision closer to the lesion and a reduced volume of healthy parenchyma sacrificed. After tumor resection, the MR model could also visualize the structures on the excision plane, facilitating precise closure of vessels and calyces in case of violation (Fig. 1). Therefore, the MR navigation optimized the outcomes of both the tumor resection and renorrhaphy reconstruction phases during LPN, resulting in reduced OT and WIT, decreased EBL, and fewer postoperative complications. Most importantly, all the above advantages translate directly to a potential improvement in the trifecta achievement, which was verified in both univariate and multivariate LRMs. In addition, similar to a previous report from Kobayashi *et al*.^6^, our findings confirmed that the MRLPN group exhibited a superior postoperative renal function, further supporting that MR navigation contributed to better postoperative renal function. This might be attributed to the following factors: (a) reduced WIT, (b) increased usage of selective clamping techniques, and (c) better preservation of healthy parenchyma resulting from precise resection.

**Figure 1:**
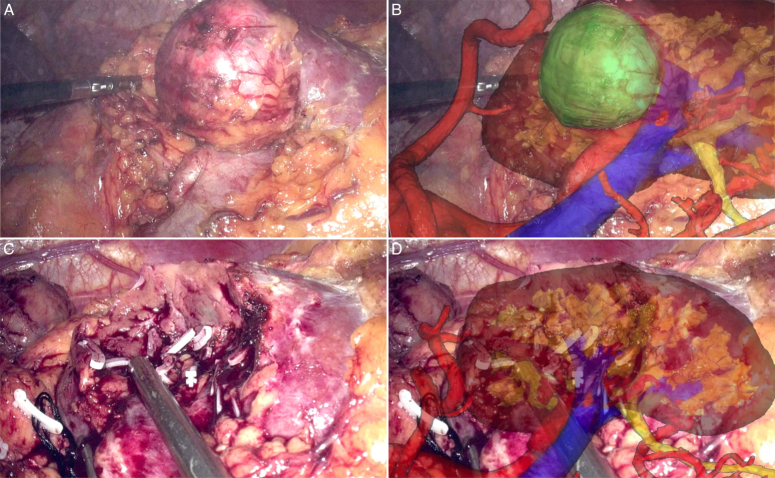
Real-time navigation with MR system in LPN. (A, C) intraoperative laparoscopic camera image. (B) overlapping of the MR model with surgical field to guide precise tumor resection. (D) after tumor resection, MR model facilitated precise closure of violated vessels and calyces on the excision plane. LPN, laparoscopic partial nephrectomy; MR, mixed reality.

The major limitation of this study is that it is a single-center trial with a relatively short follow-up period and limited sample size. Prospective randomized studies with larger sample sizes and longer follow-ups are still required to evaluate the benefit of MR navigation system.

In conclusion, our findings demonstrated the efficacy and advantages of MR navigation system in LPN procedures for highly complex tumors. Real-time surgical navigation based on MR technology has the potential to significantly enhance surgical precision during both tumor resection and renal reconstruction, thereby achieving the aim of ‘precision PN’, reducing postoperative complications, and optimizing functional recovery.

## Ethics approval

This study was approved by the Institutional Ethics Committee of Shengjing Hospital of China Medical University (No. 2021PS167J).

## Consent for publication

We obtained informed consent from each patient.

## Source of funding

This research was supported by the National Natural Science Foundation of China (No. 82173372 and No. 81802540), the Education Department Grant of Liaoning Province (No. LJKMZ20221138), and the Bethune Urologic Oncology Special Project Research Fund (No.mnzl202023).

## Author contribution

W.L., Y.W., and Z.W.: contributed equally to this work and should be considered co-first authors; W.L.: investigation, validation, and writing – original draft; Y.W. and Z.W.: writing – original draft; Z.C.: data curation and resources; Y.Y.: formal analysis; J.W. and J.B.: visualization; L.W.: conceptualization, methodology, and writing – review and editing; M.Z.: conceptualization, funding acquisition, methodology, project administration, resources, supervision, and writing – review and editing.

## Conflicts of interest disclosure

The authors declare that they have no competing interests.

## Research registration unique identifying number (UIN)

ChiCTR2400086111.

## Guarantor

Mo Zhang, Department of Urology, The First Hospital of China Medical University, Shenyang 110001, People’s Republic of China. E-mail: peterzhang623@gmail.com

## Data availability statement

The datasets used and analyzed during the current study are available from the corresponding author upon reasonable request.

## Provenance and peer review

Not commissioned, externally peer-reviewed.

## Supplementary Material
