# Supplementary material for: The application of mixed reality navigation system in laparoscopic partial nephrectomy for highly complex renal tumors (RENAL score ≥10): a retrospective cohort study
Source: Int J Surg. 2024 Aug 2;111(1):1513–6. doi: 10.1097/JS9.0000000000001983 (PMC11745591; doi:10.1097/JS9.0000000000001983)
Supplement: Supplementary file 1 [file js9-111-1513-s001.pdf]

Supplementary Materials

The application of mixed reality navigation system in laparoscopic partial nephrectomy for highly complex renal tumors (RENAL score ≥ 10): a retrospective cohort study

Content

Supplementary Methods

1. Study population
2. Three-dimensional mixed reality model rendering
3. Surgical technique
4. Data analysis
5. Statistical analysis
6. Supplementary abbreviations
7. Supplementary references

Supplementary Table 1. Patients' demographic and clinical characteristics between the MRLPN and SLPN groups.

Supplementary Figures

Supplementary Figure 1. Flowchart of patient selection for the study.

Supplementary Figure 2. Flowchart of 3D MR model rendering.

Supplementary Figure 3. The application scenario of MR model during surgery.

Supplementary Figure 4. RFS comparison between the MRLPN and SLPN groups.

Supplementary Methods

1. Study population

Patients diagnosed with highly complex renal tumors at our institution were consecutively enrolled in the study from October 2020 to March 2024. The inclusion criteria were patients who underwent laparoscopic partial nephrectomy (LPN) and had a localized renal tumor with a RENAL nephrometry score ≥10, while the exclusion criteria included patients with solitary kidney, recurrent tumor, multiple tumors, bilateral tumors or loss of follow-up. A retrospective analysis of the patients was performed, as shown in Supplementary Figure 1. For all cases, based on surgeon's expertise and preference, either mixed reality-assisted LPN (MRLPN) or standard LPN (SLPN) was performed. All LPN procedures were performed by the same experienced surgeon. We divided the patients into two groups according to whether they underwent mixed reality (MR) navigation, namely, the MRLPN and SLPN group. This study was approved by the Institutional Ethics Committee of our hospital and was conducted in compliance with the Declaration of Helsinki. We obtained informed consent from each patient. This study has been reported in accordance with STROCSS standards[1] (Supplemental Digital Content 2).

2. Three-dimensional mixed reality model rendering

All patients included in the study underwent preoperative renal contrast-enhanced computed tomography (CT) scan, and the CT data were collected and saved in Digital Imaging and Communications in Medicine (DICOM) format. Subsequently, we imported all DICOM files of the MRLPN group into holographic MR modeling software (VISUAL, Beijing, China). Using the software's segmentation and reconstruction functionalities (Supplementary Figure 2a), renal vasculature (both arteries and veins), renal tumors, kidney parenchyma, the collecting system, and other surrounding structures can be accurately mapped and reconstructed. The three-dimensional (3D) model was then saved as a stereolithography file, which was further imported into scene editing mode (Supplementary Figure 2b). In this mode, the color and transparency of target tissues could be adjusted to optimize the holographic 3D effect (Supplementary Figure 2c). Finally, the MR model was integrated into the HoloLens equipment and the laparoscopic video system.

3. Surgical technique

In the MRLPN group, the MR model was overlaid with real-time laparoscopic imaging to guide the operation (Supplementary Figure 3). The 3D visualization of renal vasculature could help the surgeon dissect the renal pedicle, followed by clamping of either the main renal artery or the tumor's feeding arteries using a bulldog technique. After dissection of the renal pedicle, MR navigation facilitated rapid and accurate localization of the tumor, which was marked on the kidney surface if it was completely endophytic. Subsequently, enucleoresection or enucleation was performed according to the preoperative planning. Intraoperative real-time MR navigation helped the surgeon evaluate the margin between the tumor and normal kidney tissues and aid in determining the optimal resection plane, which contributed to precise tumor removal. During the renorrhaphy reconstruction phase, accurate closure of the collecting system and vessels was achieved using Hem-o-lok clips combined with 3-0 V-Loc sutures under MR navigation, by rotating, hiding, and scaling the MR image. A double-layer suture of the renal parenchyma with 2-0 V-Loc sutures followed. Finally, the surgeon removed the bulldog clamp to restore renal blood supply.

In contrast, surgical planning for the SLPN group was based solely on preoperative contrast-enhanced CT scan, followed by conventional LPN under the guidance of intraoperative ultrasonography.

4. Data analysis

For each patient, we retrospectively collected demographic data (including age, gender, body mass index, comorbidities classified according to the Charlson comorbidity index[2], and American Society of Anesthesiologist score); clinical characteristics (including tumor size, side, complexity of tumors by RENAL nephrometry score[3], Mayo Adhesive Probability score, and clinical TNM stage[4]); perioperative data (including surgical approach, operation time, warm ischemia time (WIT), estimated blood loss, transfusion rate, and length of postoperative hospital stay); pathological data (including rate of positive surgical margin, pathological stage and pathological type); and renal-functional outcomes (including serum creatinine [SCr] level and estimated glomerular filtration rate [eGFR]). The SCr and eGFR were recorded preoperatively and 3 months post-surgery, respectively. We calculated eGFR using the CKD-EPI equation[5] from SCr. Postoperative complications were classified according to the Clavien–Dindo system[6]. Patients diagnosed with renal cell carcinoma via pathological examination underwent oncological follow-

up, which was composed of laboratory evaluation and imaging examination. The former was conducted at 3, 6, 12, 18, and 24 months after surgery, and annually thereafter, the latter at 6, 12, 18, and 24 months and annually thereafter.

To evaluate the two different techniques, we compared perioperative and pathological data as well as renal-functional and oncological outcomes during follow-up. In addition, we assessed the optimal surgical outcomes defined as the achievement of trifecta (WIT < 25 min, negative surgical margins, and no perioperative complications)[7].

5. Statistical analysis

Statistical analysis were conducted using SPSS version 22.0 (IBM Corp., Armonk, NY, USA). Continuous variables with normal distributions were reported as means and standard deviations, while those not following a normal distribution were presented as medians and interquartile ranges. Categorical variables were described in terms of frequencies and proportions. Independent-sample Student's t-test was used to compare mean values of normally distributed continuous variables, whereas the Mann–Whitney U test was employed for non-normally distributed continuous variables. Categorical variables were compared using either the $\chi 2$ test or Fisher's exact test. Univariate and multivariate logistic regression models were conducted to determine the predictors of optimal surgical outcomes, reported as the odds ratios and corresponding 95% confidence intervals. Kaplan–Meier survival curves were drawn to assess recurrence and survival. Statistical significance was defined as $P < 0.05$.

6. Supplementary abbreviations

LPN, laparoscopic partial nephrectomy; MRLPN, mixed reality-assisted LPN; SLPN, standard LPN; MR, mixed reality; CT, computed tomography; DICOM, Digital Imaging and Communications in Medicine; 3D, three-dimensional; WIT, warm ischemic time; SCr, serum creatinine; eGFR, estimated glomerular filtration rate.

7. Supplementary references

[1] Mathew G, Agha R, Albrecht J, *et al*. STROCSS 2021: Strengthening the reporting of cohort, cross-sectional and case-control studies in surgery. Int J Surg 2021;96:106165.

[2] Nuttall M, van der Meulen J, Emberton M. Charlson scores based on ICD-10 administrative data were valid in assessing comorbidity in patients undergoing urological cancer surgery. J Clin Epidemiol 2006;59:265-273.

[3] Kutikov A, Uzzo RG. The R.E.N.A.L. nephrometry score: a comprehensive standardized system for quantitating renal tumor size, location and depth. J Urol 2009;182:844-853.

[4] Moch H, Artibani W, Delahunt B, *et al*. Reassessing the current UICC/AJCC TNM staging for renal cell carcinoma. Eur Urol 2009;56:636-643.

[5] Levey AS, Stevens LA, Schmid CH, *et al*. A new equation to estimate glomerular filtration rate. Ann Intern Med 2009;150:604-612.

[6] Dindo D, Demartines N, Clavien PA. Classification of surgical complications: a new proposal with evaluation in a cohort of 6336 patients and results of a survey. Ann Surg 2004;240:205-213.

[7] Khalifeh A, Autorino R, Hillyer SP, *et al*. Comparative outcomes and assessment of trifecta in 500 robotic and laparoscopic partial nephrectomy cases: a single surgeon experience. J Urol 2013;189:1236-1242.

Supplementary Table 1. Patients' demographic and clinical characteristics between the MRLPN and SLPN groups.

Variables	MRLPN group	SLPN group	*P* value
Number of patients	48	60	
Males, n (%)	28 (58.3)	34 (56.7)	0.862
Age (y), mean (SD)	59.1 (9.5)	58.0 (9.8)	0.552
BMI (kg/m^2), median (IQR)	22.9 (21.0–23.6)	22.9 (20.9–23.6)	0.916[a]
CCI, median (IQR)	0 (0–0)	0 (0–0)	0.216[a]
CCI age-adjusted, median (IQR)	1.5 (1–2)	2 (1–2)	0.930[a]
ASA score, median (IQR)	2 (2–2)	2 (2–2)	0.830[a]
Tumor left side, n (%)	27 (56.3)	28 (46.7)	0.322
Tumor size (mm), mean (SD)	54.3 (17.3)	52.8 (16.0)	0.640
RENAL score, median (IQR)	11 (10–11)	10 (10–11)	0.212[a]
MAP score, median (IQR)	3 (3–4)	3 (3–4)	0.141[a]
Clinical stage, n (%)			0.523
T1a (≤ 4cm)	10 (20.8)	12 (20.0)	
T1b (4 cm < and ≤ 7 cm)	26 (54.2)	38 (63.3)	
T2a (7 cm < and ≤ 10 cm)	12 (25.0)	10 (16.7)	
Pathological stage, n (%)			0.573
pT1a	9 (18.8)	10 (16.7)	
pT1b	23 (47.9)	34 (56.7)	
pT2	10 (20.8)	7 (11.7)	
pT3	6 (12.5)	9 (15.0)	
Pathological type, n (%)			1.000[b]
Clear-cell RCC	42 (87.5)	53 (88.3)	
Papillary RCC	4 (8.3)	5 (8.3)	
Chromophobe RCC	2 (4.2)	2 (3.3)	
Preoperative eGFR (ml/min/1.73 m^2), mean (SD)	95.8 (12.9)	98.3 (13.7)	0.328

LPN, laparoscopic partial nephrectomy; MRLPN, mixed reality–assisted LPN; SLPN, standard LPN; SD, standard deviation; BMI, body mass index; IQR, interquartile range; CCI, Charlson Comorbidity Index; ASA score, American Society of Anesthesiologists score; MAP score, Mayo Adhesive Probability score; RCC, renal-cell carcinoma; eGFR, estimated glomerular filtration rate. [a]: Mann–Whitney *U* test; [b]: Fisher's exact test.

Supplementary Figure 1. Flowchart of patient selection for the study.

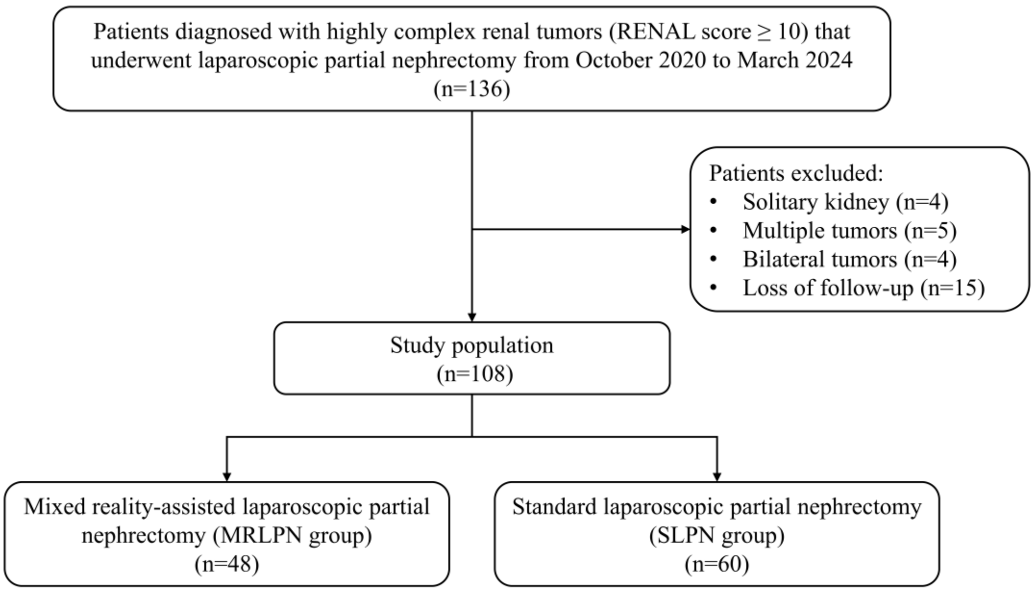

Supplementary Figure 2. Flowchart of 3D MR model rendering.

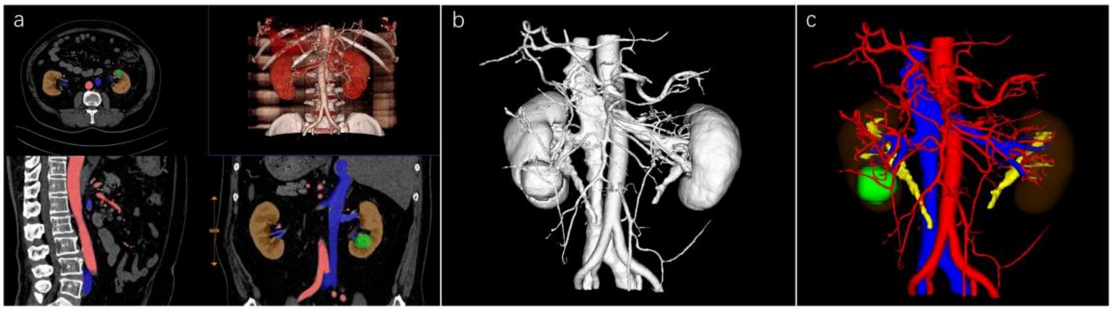

Supplementary Figure 3. The application scenario of MR model during surgery.

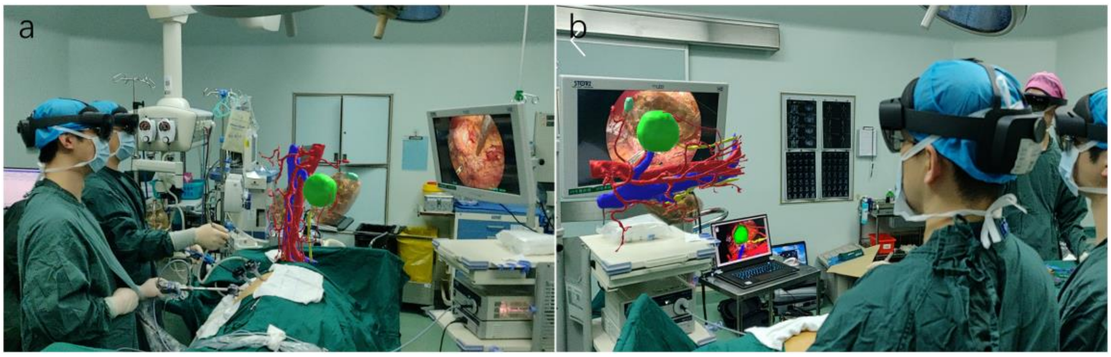

Supplementary Figure 4. RFS comparison between the MRLPN and SLPN groups.

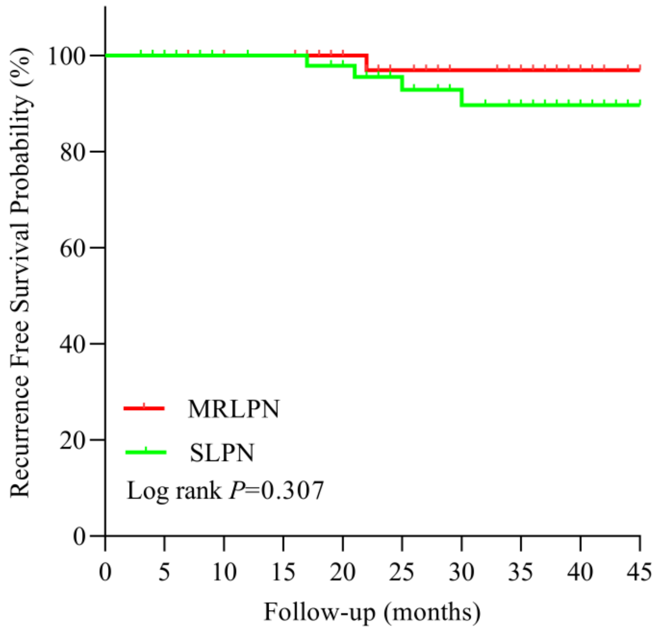

Supplementary Figure legends

Supplementary Figure 1. Flowchart of patient selection for the study.

Supplementary Figure 2. Flowchart of 3D MR model rendering. (a) segmentation and reconstruction stage. (b) scene editing mode. (c) final established holographic 3D model. 3D, three-dimensional; MR, mixed reality.

Supplementary Figure 3. The application scenario of MR model during surgery. (a) MR model was superimposed onto the real-world setting. (b) MR model was overlaid with laparoscopic imaging. MR, mixed reality.

Supplementary Figure 4. RFS comparison between the MRLPN and SLPN groups. RFS, recurrence-free survival; MRLPN, mixed reality–assisted LPN; SLPN, standard LPN.
